# Partial *Treponema* spp. as candidate probiotics for rumen methane mitigation revealed by a module-based activity index

**DOI:** 10.3389/fvets.2025.1654829

**Published:** 2025-09-05

**Authors:** Wei Wang, Xiaoyun Chen, Donghui Fang, Linxiang Li, A. G. Yueda, Jia Gan, Xiaodong Deng, Xiaoqin Ma, Ying Chen, Yi Shi, Fang He, Changfeng Wu, Zhixin Yi, Maozhong Fu, Jun Yi

**Affiliations:** ^1^Animal Breeding and Genetics Key Laboratory of Sichuan Province, Sichuan Animal Science Academy, Chengdu, China; ^2^Bazhong Academy of Agriculture and Forestry Sciences, Bazhong, China

**Keywords:** methanogenesis pathway, archaea–bacteria interactions, rumen methane mitigation, meta-transcriptome, probiotics

## Abstract

**Introduction:**

Methane emissions from ruminants, driven by methanogenic archaea, are a major source of greenhouse gases. Current strategies often rely on metagenomic (MG) abundance as a proxy for methanogenic potential, despite evidence of a disconnect with *in-situ* activity.

**Methods:**

We analyzed paired MG and meta-transcriptomic (MT) datasets from 48 bovine rumen samples. Comparative analyses were performed to assess microbial taxonomic abundance versus transcriptional activity. A Methanogenesis Pathway Expression Activity Index (MPEAI) was developed by integrating expression of four KEGG modules, and Random Forest modeling was applied to identify microbial taxa associated with MPEAI.

**Results:**

MG and MT profiles showed incongruence in both microbial community composition and diversity, with MT revealing reduced archaeal transcriptional activity. Dominant archaeal genera (*Methanobrevibacter*, *Methanocaldococcus*) were transcriptionally suppressed relative to MG abundance (*p* < 0.001). In contrast, methanogenesis modules (M00356, M00567, M00357, M00563) exhibited higher expression in MT than MG (*p* < 0.0001), indicating pathway-level hyperactivity despite archaeal suppression. Random Forest analysis linked MPEAI variation to several *Treponema* species, which showed significant negative correlations with methanogenic pathway activity (*r* = −0.36 to −0.57, *p* < 0.01).

**Conclusion:**

Rumen methanogenesis is regulated by functional pathway activity rather than archaeal abundance. The consistent negative associations of *Treponema* species with methanogenesis highlight their potential as probiotic candidates for methane mitigation and underscore bacterial-archaeal interactions in shaping rumen methane production.

## 1 Introduction

Archaea represents a significant component of the gut microbiota, with established roles in, host health (1, 2), and nutrient metabolism (3, 4). Previous research demonstrates that archaea engage in dynamic interrelationships with bacteria and fungi within the intestinal ecosystem (5), and contribute to the stability of the gut environment. Rumen methanogenesis, such as *Methanobrevibacter*, *Methanocaldococcus*, and *Methanosarcina*, an essential microbial process in ruminants, converts multiple substrates—including hydrogen (H₂), carbon dioxide (CO₂), acetate, methanol, and methylamines—into methane (CH₄) (6–8). This process not only supports anaerobic digestion but also contributes to global greenhouse gas emissions (9). Methane has global warming potential 28–36 times higher than CO₂ over a century (10, 11). Methane emissions from ruminant livestock constitute a substantial portion of anthropogenic greenhouse gases, acting as a major driver of climate change (12). Therefore, understanding and mitigating rumen methane production while maintaining animal productivity and health is critical research.

As a core method in current gut microbiome studies, metagenomic sequencing is widely used to reconstruct microbial genomes, microbial diversity and analyze functional diversity (13–15). Current methane mitigation strategies predominantly rely on methanogen abundance derived from metagenomic profiling as a proxy for methanogenic potential. However, emerging evidence reveals a fundamental disconnect between genomic abundance and in-situ functional activity (16, 17). For instance, in sheep with contrasting methane yield phenotypes, meta-transcriptomic expression of hydrogenotrophic methanogenesis pathway genes was significantly higher in high-methane yield animals—even when the corresponding metagenomic abundance showed no significant differences (18). The extent to which MT of core methanogens and pathways align with their MG abundance in cattle rumen remains unresolved (16, 19). Recent studies have highlighted probiotics as promising agents for mitigating methane emissions in ruminants, focusing particularly on lactic acid bacteria (LAB) and propionate-producing bacteria (PAB) (20, 21). Species such as *Lactiplantibacillus plantarum*, *Ligilactobacillus ruminis*, and *Lactobacillus amylovorus* have been explored due to their ability to alter fermentation patterns and reduce hydrogen availability to methanogenic archaea (22). Additionally, *Megasphaera elsdenii*, *Selenomonas ruminantium*, and Acidipropionibacterium thoenii demonstrate efficacy by promoting propionate pathways, thereby diverting hydrogen away from methane production (21). Despite promising *in vitro* results, *in vivo* applications remain inconsistent due to strain persistence issues, variable dosage efficacy, and interactions with host microbiota and diets (22). Meta-analysis indicates multi-strain probiotics outperform single-strain supplements, but practical implementation continues to face significant challenges, including strain selection, dosage optimization, and context-dependent effectiveness (23). Therefore, identifying novel probiotic candidates with targeted functions, such as fiber-degrading capacity and hydrogen-modulation potential, is crucial.

This study aims to reveal the relationship between rumen microbial community composition (taxonomic abundance) and the functional gene expression of core methanogenesis pathways, while identifying key microbial taxa associated with gene expression associating with active methane production. To address the critical gap in linking genomic potential to in-situ functional activity, we introduce the MPEAI, integrating the coordinated expression of four central KEGG modules: hydrogenotrophic (M00567), methylotrophic (M00356), acetoclastic (M00357), and cofactor synthesis (M00563) modules. Leveraging paired metagenomic (MG) and meta-transcriptomic (MT) datasets, our integrated analytical strategy: (1) quantifies taxonomic abundance and pathway expression; (2) compares genomic abundance versus transcriptional activity to identify functional discrepancies; and (3) applies Random Forest modeling to pinpoint microbial drivers of methanogenic pathway activity.

## 2 Materials and methods

### 2.1 Data collection and pre-processing

The study incorporated 48 bovine rumen microbial samples encompassing both metagenomic and meta-transcriptomic sequencing datasets (24). The raw sequencing dataset was obtained from the NCBI Sequence Read Archive (SRA) under accession number PRJNA393057. Raw sequencing data underwent preprocessing through the Kneaddata pipeline (v0.7.2) with three critical phases: Quality trimming and adapter removal were initially performed using Trimmomatic (v0.39) (25), followed by host-derived sequence elimination through alignment against the bovine reference genome (GenBank accession: GCF_002263795) via Bowtie 2 (Version 2.5.3) (26). To address elevated ribosomal RNA (rRNA) abundance in meta-transcriptomic profiles, SortMeRNA (v4.3.2) (27) paired with the SMR v4.3 refined database were used to remove rRNA sequences from both data types, thereby mitigating analytical bias in subsequent selected probiotic taxon expression quantification. The resultant high-fidelity cleaned reads served as the foundation for downstream bioinformatic exploration.

### 2.2 Quantification of methanogenic pathway activity

The gene abundance and expression were quantified using salmon (v1.3.2; option l A). Methanogenic pathway activity was quantified using a weighted Z-score approach based on metagenomic sequencing data. Genes with transcript per million (TPM) values > 1 in ≥ 10% of samples were retained to ensure robust expression detection. Core methanogenesis-related genes were identified through KEGG module annotation (M00567, M00357, M00356, M00563) using eggNOG database annotations and eggnog-mapper (28).

A dual normalization strategy was employed: gene expression levels were standardized across samples using Z-score transformation:

$$Z_{g}=\frac{E_{g,s}-\mu_{g}}{\sigma_{g}}$$

where $E_{g,s}$ denotes the transcript-per-million (TPM) value of gene g in s sample, and $\mu_{g}$ (mean) and $\sigma_{g}$ (standard deviation) were calculated across all samples. This step minimized technical biases arising from heterogeneous expression scales. Subsequently, inverse variance weights ( $w_{g}$ ) were assigned to prioritize genes with stable expression patterns:

$$w_{g}=\frac{1}{\sigma_{g}^{2}+\in }\;(\in =10-6\;\text{to avoid division}\;\mathrm{by}\;\text{zero}).$$

Weighted pathway activity for each sample was then computed by integrating Z-scores and weights across all annotated genes in the methanogenesis modules:


$$A_{s}=\frac{\sum_{g\in }Z_{g,s}\cdot w_{g}}{\sum_{g\in }w_{g}}$$

where denotes the set of pathway-associated genes. Finally, cross-sample normalization was performed to ensure comparability:


$$\tilde{A_{s}}=\frac{A_{s}-\mu_{A}}{\sigma_{A}}$$

where $\mu_{A}$ and $\sigma_{A}$ are the mean and standard deviation of raw activity scores across all samples. This approach integrates coordinated expression patterns of pathway-associated genes while enhancing statistical robustness through variance-sensitive weighting.

### 2.3 Probiotic taxonomy profiling and diversity calculation

Taxonomic classification of metagenomic and meta-transcriptomic data was performed using Kraken2 (v2.1.2) (29) with parameter of “--paired.” Clean reads were aligned against archaeal reference genomes from the Genome Taxonomy Database (GTDB release 207) (30) which was pre-processed via the Struo2 pipeline (31) with default parameters prior to analysis.

Taxonomic quantification data of archaeal and bacterial species were processed through the QIIME2 pipeline (2024.5) (32). Sequence reads underwent rarefaction to normalize sampling depth, followed by calculation of relative abundance for archaeal and bacterial communities. To assess archaeal diversity, a rarefied species-level count matrix was re-imported into QIIME2 for alpha diversity (Shannon Index).

### 2.4 Statistical validation

Random Forest Model for Identifying Key Microbes Associated with Methanogenic Pathway Activity with the following key parameters: mtry = 3, and ntree = 10,000. Between-group differences in diversity metrics were evaluated using the Kruskal-Wallis nonparametric test, while community structure variation was tested via Analysis of Similarities (ANOSIM). Statistical significance was defined at *p* ≤ 0.05 for all analyses. Data visualization was implemented with the ggplot2 package in R (v4.3.2).

## 3 Results

### 3.1 Incongruence between genomic abundance and transcriptional activity in rumen methanogens

**Figure 1:**
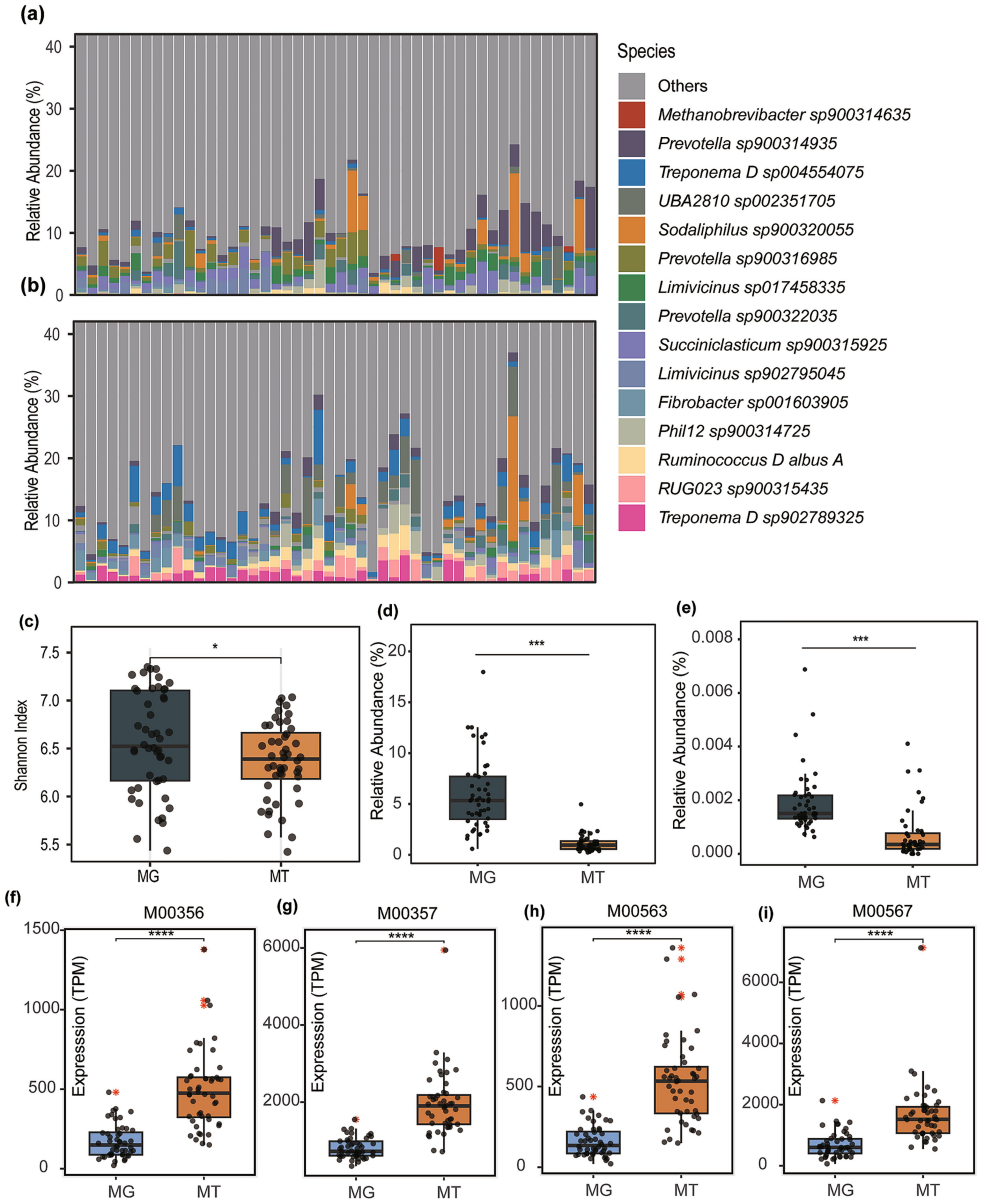
Microbial community composition and functional comparisons between metagenomic (MG) and metatranscriptomic (MT) datasets. **(a)** boxplot displaying the top 15 microbial taxa ranked by mean relative abundance in metagenomic profiles. **(b)** Corresponding taxonomic distribution derived from metatranscriptomic data. **(c)** Comparative analysis of species-level alpha diversity (Shannon index) between MG and MT samples. **(d)** Differential abundance of the genus Methanobrevibacter across MG and MT datasets. **(e)** Relative abundance variations of Methanocaldococcus genus between MG and MT profiles. Abundance and expression of methanogenesis-associated modules was compared, including Module M00356 **(f)**, M00357 **(g)**, M00563 **(h)**, and M00567 **(i)**. Asterisks denote statistical significance determined by Wilcoxon (*p < 0.05, ***p < 0.001, ****p < 0.0001).

To investigate the relationship between rumen microbial communities and methanogen expression activity in cattle, paired metagenomic and meta-transcriptomic datasets from 48 cattle in Li et al.’s study were analyzed. Taxonomic profiling using Kraken2 (v2.1.2) and the GTDB (release 207) database revealed incongruence between the relative abundance rankings of microbial species in MG and MT datasets (Figures 1a,b). The top five species by metagenomic relative abundance were *Methanobrevibacter sp900314635* (3.14%), *Prevotella sp900314935* (2.08%), *Prevotella sp900316985* (1.36%), *Succiniclasticum sp900315925* (1.29%), and *Sodaliphilus sp900320055* (1.20%). In contrast, the metatranscriptomic profiles prioritized *Treponema D sp004554075* (1.97%), *UBA2810 sp002351705* (1.78%), *RUG023 sp900315435* (1.24%), *Fibrobacter sp001603905* (1.24%), and *Treponema D sp902789325* (1.19%). Notably, most highly abundant taxa were assigned GTDB-specific identifiers, indicative of uncultured microbial lineages, suggesting a substantial reservoir of uncultivated microorganisms in the bovine rumen.

Comparative analysis of species-level alpha diversity (Shannon index) demonstrated significantly higher diversity in MG compared to MT datasets (Wilcoxon rank-sum test, *p* < 0.05; Figure 1c). Previous studies, such as Peng et al., have identified archaeal taxa (e.g., *Methanobrevibacter* spp.) with high transcriptional activity in domesticated animals. However, the current findings suggest an overall transcriptional suppression state within the rumen microbial community of cattle.

Archaea, recognized as the primary methanogenic microorganisms, are dominated in the bovine gut by genera such as *Methanobrevibacter* and *Methanocaldococcus* according to prior studies. Comparative analysis of these genera revealed markedly higher relative abundances in metagenomic profiles compared to their transcriptional activity in meta-transcriptomic (MT) datasets (*p* < 0.001; Figures 1d,e). Specifically, *Methanobrevibacter* exhibited a metagenomic abundance of 5.91% (MG) versus 1.11% (MT; Figure 1d), while *Methanocaldococcus* showed 0.0019 (for MG) *vs.* 0.00070% (for MT; Figure 1e). This pronounced disparity underscores a systemic transcriptional suppression of methanogenic archaea within the rumen microbial community, aligning with the observed overall reduction in microbial expression activity.

Based on meta-transcriptomic profiling, this study observed that although the overall functional gene expression of dominant methanogens was suppressed, significantly enhanced activity was detected in key methanogenesis-associated metabolic modules. Specifically, the total expression levels of four critical modules in meta-transcriptomes exhibited statistically higher values (*p* < 0.0001) than their relative abundance in metagenomes: M00356 [Methyl-coenzyme M reductase, core methanogenesis; TPM (MT) = 528.16 vs. TPM (MG) = 174.71; Figure 1f], M00357 [Tetrahydromethanopterin S-methyltransferase, hydrogenotrophic pathway; TPM (MT) = 1647.40 vs. TPM (MG) = 746.32; Figure 1g], M00563 (Acetyl-CoA decarbonylase synthase, acetoclastic pathway; TPM (MT) = 532.78 vs. TPM (MG) = 160.07; Figure 1h), and M00567 (Coenzyme M biosynthesis, methanogen cofactor synthesis; TPM (MT) = 1940.07 vs. TPM (MG) = 808.02; Figure 1i).

### 3.2 Identification of potential methane-mitigating probiotics via functional gene expression profiling

Previous studies have often analyzed methanogen abundance as a proxy for methanogenic potential. However, our analysis revealed that methanogen abundance often appeared lower than, or did not consistently correlate with, the functional expression of methanogenesis pathways. Conversely, the expression of genes comprising key methanogenesis-related metabolic modules demonstrated robust activity. Therefore, we established the Methanogenesis Pathway Expression Activity Index based on the gene expression levels of four key KEGG modules associated with methanogenesis (M00567, M00357, M00356, and M00563). This index was used to identify microbial species associated with the activity of these methanogenic pathways.

Random Forest model was employed to identify microbial species explaining variance in MPEAI (Supplementary Figure S1). Among the top 30 species ranked by feature importance, only five corresponded to currently known cultivated species, while the remaining 25 were represented solely by Genome Taxonomy Database (GTDB) identifiers (indicating they are yet-uncultivated). Pearson correlation analysis revealed that 11 of these species exhibited significant negative correlations with MPEAI. These included *UBA1240 sp016285185*, *Treponema D succinifaciens*, and nine other yet-uncultivated *Treponema* species (Supplementary Figure S2).

**Figure 2:**
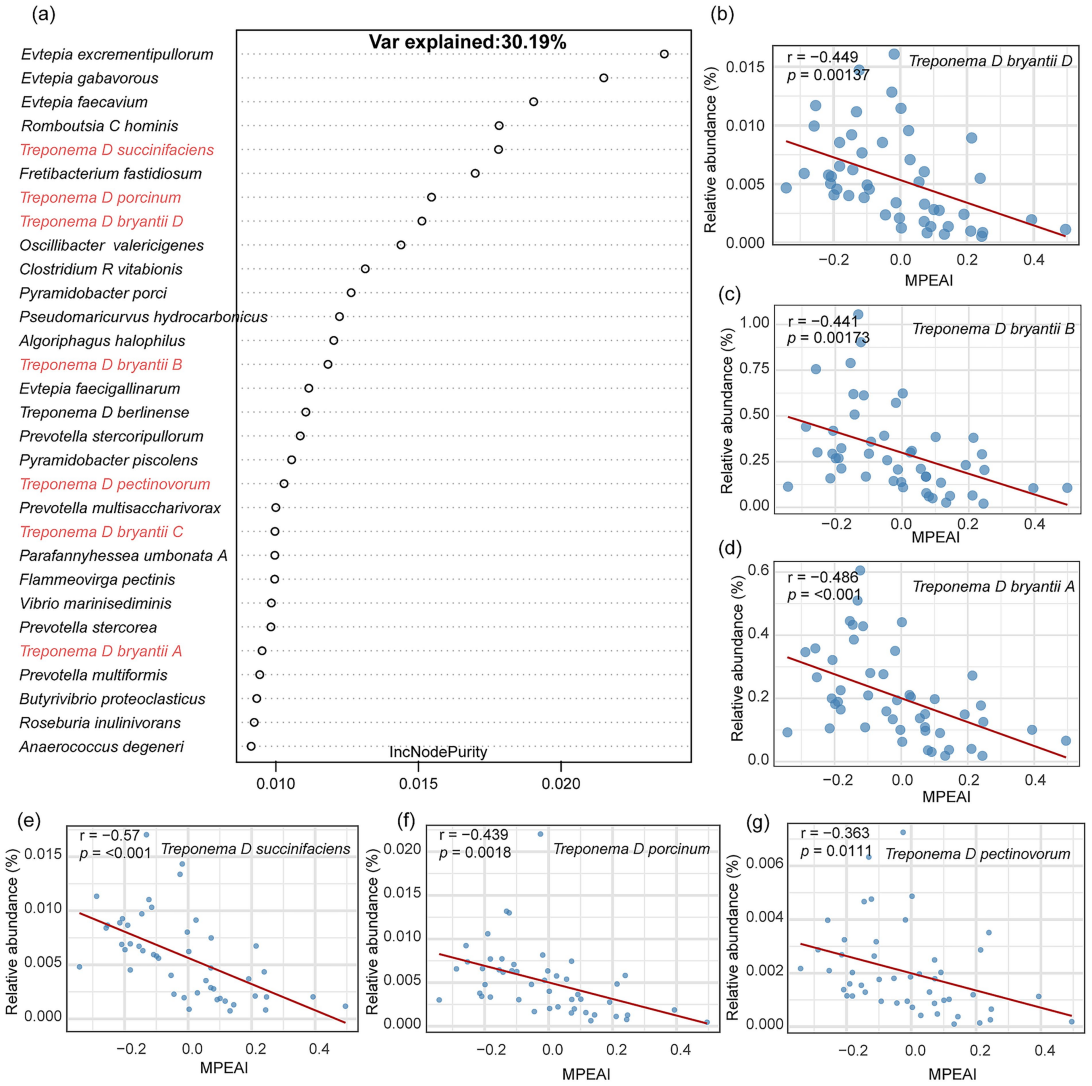
Identification of key rumen microorganisms associated with the methanogenesis index using a random forest model. **(a)** Top 30 important bacterial species identified by the random forest model based on lncNodepurity importance ranking. **(b–g)** Scatter plots demonstrate a significant negative correlation between the species *Treponema* spp. and the methanogenesis index. *r* represents the Pearson correlation coefficient. y-axis represents the relative abundance of corresponding species. x-axis represents the Methanogenesis Pathway Expression Activity Index (MPEAI).

Due to the prevalence of potentially taxonomically unresolved (as indicated by GTDB identifiers) and currently uncharacterized species among the uncultivated organisms identified, which precludes functional follow-up, we excluded uncultivated species and repeated the Random Forest analysis. Following the exclusion of uncultivated taxa, the Random Forest model identified the three species classified under the genus Evtepia as the top features positively associated with MPEAI (Figure 2a). Species exhibiting significant negative correlations with MPEAI included: *Treponema D bryantii D* (Figure 2b; R = − 0.449, *p* = 0.00137), *Treponema D bryantii B* (Figure 2c; R = − 0.441, *p* = 0.0013), and *Treponema D bryantii A* (Figure 2d; R= − 0.486, *p* < 0.001), *Treponema D succinifaciens* (Figure 2e; R = − 0.570, *p* < 0.001), *Treponema D porcinum* (Figure 2f; R = − 0.439, *p* = 0.0018), and *Treponema D pectinovorum* (Figure 2g; R = − 0.363, *p* = 0.0111). These results indicate a consistent negative association between members of the genus *Treponema* and MPEAI, suggesting their potential role as probiotics for mitigating methane production.

## 4 Discussion

Rapid global population growth necessitates a 73% increase in milk and meat production by 2050 to satisfy rising food demand (33). Simultaneously, humanity must urgently address the critical challenge of curbing escalating greenhouse gas emissions to mitigate climate change, especially for ruminants (34). Developing effective strategies to reduce livestock emissions requires a deeper understanding of the rumen microbes responsible for methane production. Our study, revealing a critical decoupling between metagenomic potential and functional activity in rumen methanogens, contributes to this understanding. While dominant archaeal genera (*Methanobrevibacter*, *Methanocaldococcus*) exhibited significantly higher metagenomic abundance than meta-transcriptomic activity (*p* < 0.001), key methanogenesis KEGG modules (M00356, M00357, M00563 and M00567) showed 2–3 × higher relative abundance in MT versus MG (*p* < 0.0001; Figures 1d–i). Our results align partially with Peng et al.’s reports of variable archaeal activity in domesticated ruminants but extend beyond them by quantifying pathway-level resilience (35). This paradox—archaeal suppression coexisting with pathway hyperactivity—suggests two potential mechanisms: (1) functional redundancy in non-archaeal taxa expressing methanogenesis modules, such as *Prevotella* (36); and (2) During host-microbe coevolution, methanogens enhance their energy acquisition efficiency by upregulating key methanogenesis pathway genes (e.g., mcrA, frhA) through adaptive evolutionary mechanisms, thereby optimizing the nutritional and metabolic adaptability of the host animal. However, our current dataset lacks the analytical resolution to differentiate the underlying causes of this phenomenon.

Here, Random Forest modeling identified *Treponema* species (e.g., *T. succinifaciens*, r = −0.570, *p* < 0.001) as consistent negative correlation of MPEAI. Here, we extend this observation to the cattle rumen, providing the evidence of such an association in this host system. *Treponema*, identified as significant rumen spirochetes in previous studies (37), participates in fiber degradation through interactions with fibrolytic bacteria (38). Li et al.’s research revealed a significant negative correlation between methanogenic archaea (order *Methanoplasmatales*) and *Treponema* species in the rumen of sika deer (39). Comparable negative correlations between *Treponema* species and methanogenic archaea have been documented in human oral microbiome studies (40). Here, we identified a significant negative correlation between abundant *Treponema* species and methanogenesis-related metabolic modules. The underlying mechanism involves *Treponema* species employing hydrogen-dependent CO₂ fixation to synthesize acetate via the acetyl-CoA pathway, thereby suppressing methanogenic pathway activity through substrate competition (41). Thus, *Treponema* species represent promising microbial agents for targeted methane mitigation in the bovine rumen. Notably, our findings revealed that not all *Treponema* species exhibited significant inverse correlations with the MPEAI, while uncultured lineages (e.g., GTDB-classified *Treponema* sp. D) dominated the top MPEAI-correlated taxa (25/30 species). Future isolation and cultivation of these *Treponema* species are essential for experimental validation.

This study quantified relative abundance and gene expression using metagenomic and meta-transcriptomic data. However, quantification inaccuracies arose from ambiguous alignments among highly homologous genomes (42), such as those of *Treponema* species, representing a key methodological limitation. Subsequent efforts should prioritize functional screening of the 30 methanogenesis-modulating *Treponema* species identified herein, aiming to isolate empirically validated probiotic strains or identify variants with enhanced efficacy.

## 5 Conclusion

This study demonstrates that rumen methanogenesis is driven by functional pathway activity rather than archaeal abundance, as evidenced by significant transcriptional suppression of dominant methanogens alongside hyperactivity of core methanogenesis pathways. We identified *Treponema* species as robust negative correlates of pathway activity. These findings redefine methane production as a community-regulated trait mediated by bacterial-archaeal synergy, challenging archaeal-centric mitigation paradigms.

## Data Availability

The original contributions presented in the study are included in the article/Supplementary material, further inquiries can be directed to the corresponding author.
