# Supplementary figures and images for: Partial Treponema spp. as candidate probiotics for rumen methane mitigation revealed by a module-based activity index
Source: Front Vet Sci. 2025 Sep 5;12:1654829. doi: 10.3389/fvets.2025.1654829 (PMC12446044; doi:10.3389/fvets.2025.1654829)

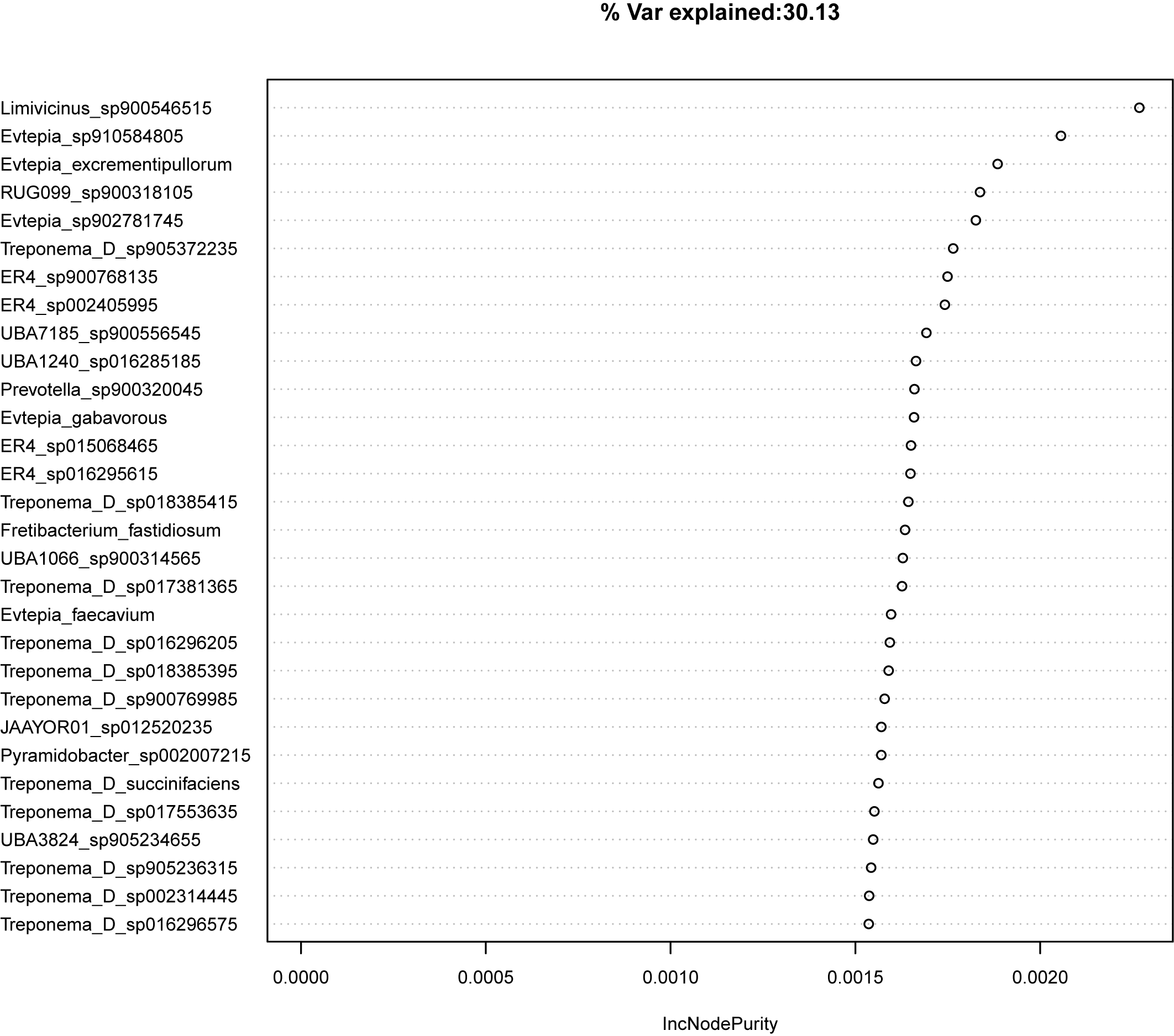

Supplement: Supplementary file 1 [file Image_1.TIF]

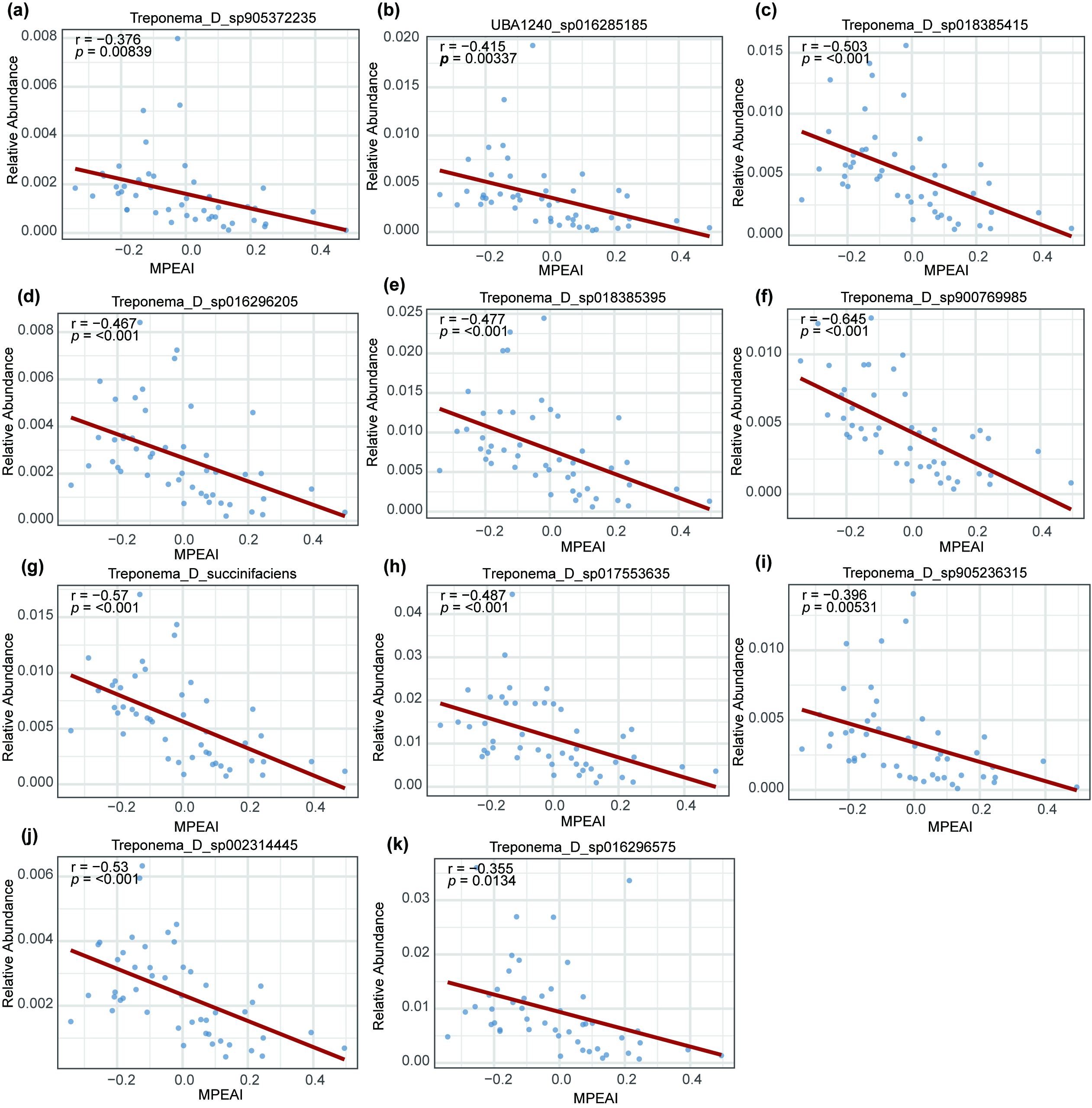

Supplement: Supplementary file 2 [file Image_2.TIF]
